# Phosphatase inhibitor 2 promotes acetylation of tubulin in the primary cilium of human retinal epithelial cells

**DOI:** 10.1186/1471-2121-9-62

**Published:** 2008-11-26

**Authors:** Weiping Wang, David L Brautigan

**Affiliations:** 1 Center for Cell Signaling, University of Virginia, School of Medicine, Charlottesville, Virginia, 22908, USA; 2 Department of Microbiology, University of Virginia, School of Medicine, Charlottesville, Virginia, 22908, USA

## Abstract

**Background:**

Primary cilia are flagella-like projections from the centriole of mammalian cells that have a key role in cell signaling. Human diseases are linked to defects in primary cilia. Microtubules make up the axoneme of cilia and are selectively acetylated and this is thought to contribute to the stability of the structure. However, mechanisms to regulate tubulin acetylation in cilia are poorly understood.

**Results:**

Endogenous phosphatase inhibitor-2 (I-2) was found concentrated in cilia of human epithelial cells, and was localized to cilia early in the process of formation, prior to the full acetylation of microtubules. Knockdown of I-2 by siRNA significantly reduced the acetylation of microtubules in cilia, without a net decrease in whole cell tubulin acetylation. There was a reduction in the percentage of I-2 knockdown cells with a primary cilium, but no apparent alteration in the cilium length, suggesting no change in microtubule-based transport processes. Inhibition of either histone deacetylases with trichostatin A, or protein phosphatase-1 with calyculin A in I-2 knockdown cells partially rescued the acetylation of microtubules in cilia and the percentage of cells with a primary cilium.

**Conclusion:**

The regulatory protein I-2 localizes to the primary cilium where it affects both Ser/Thr phosphorylation and is required for full tubulin acetylation. Rescue of tubulin acetylation in I-2 knockdown cells by different chemical inhibitors shows that deacetylases and phosphatases are functionally interconnected to regulate microtubules. As a multifunctional protein, I-2 may link cell cycle progression to structure and stability of the primary cilium.

## Background

Cilia are projections from the surface of cells that are similar to flagella. The axoneme of a primary cilium is made up of microtubules. Each cilium (and flagellum) grows out from, and remains attached to, a basal body, which is the maternal centriole [1]. Almost every cell in vertebrates has a single primary cilium with a 9+0 arrangement [2], which lacks the central pair of microtubules seen in the 9+2 arrangement of flagella and motile cilia. Primary cilia are essential for several critical signaling pathways, sensory reception and detection of fluid flow across epithelia. Recent studies suggest that a variety of human syndromes are related to defects in the assembly, maintenance and function of the primary cilium, including renal dysfunction, diabetes, and retinal degeneration [3,4].

Little is currently known about the control of the formation and resorption of cilia, although many proteins have been defined as ciliary structural components or cilia-associated signaling proteins. Several lines of evidence indicate a relationship between cell cycle and primary cilium assembly. Ciliary disassembly in many cells precedes entry into the cell cycle and ciliary assembly follows exit from mitosis [5,6]. Some proteins, such as NIMA-related kinase [7] and Aurora kinase [8,9], have been shown to play roles in both cell cycle regulation and assembly of cilia.

Protein phosphatase 1 (PP1) is a major protein Ser/Thr phosphatase with a variety of cellular functions [10-12]. PP1 exists in cells as a set of distinctive multisubunit holoenzymes [12,13], which are comprised of a PP1 catalytic subunit paired with a regulatory subunit. There are predicted to be 200+ regulatory subunits that control PP1 holoenzyme subcellular localization, catalytic activity and substrate specificity. Protein phosphatase inhibitor-2 (I-2) is a heat stable protein capable of selectively inhibiting the PP1 catalytic subunit [14]. Recent studies show that the function of I-2 is important for the cell cycle regulation and I-2 is localized at centrosomes during interphase [15]. The expression level of I-2 fluctuates during the cell cycle and is enhanced at mitosis [16], when it becomes phosphorylated at PXTP site [17,18]. Knockdown of I-2 by RNAi in mammalian cells leads to failure of cytokinesis and formation of multinucleated cells, probably due to an imbalance of Aurora B vs. PP1 [19]. During Drosophila early embryogenesis, maternal I-2 is required for proper chromosome segregation and mitotic synchrony [20]. The concept is that I-2 selectively targets certain PP1 holoenzymes to control kinase/PP1 balance and thereby trigger cellular events.

Here we report that endogenous I-2 is localized in the primary cilium of human retinal epithelial ARPE-19 cells. During the process of cilium formation, I-2 was concentrated in the cilium before axonemal tubulin was acetylated. Knockdown of I-2 by RNAi specifically reduced tubulin acetylation in the primary cilium, not the rest of the cell, and this could be rescued by chemical inhibition of either PP1 or HDAC. Our results indicate that I-2 has a role in regulation of tubulin acetylation in the primary cilium.

## Results

### Localization of I-2 in the primary cilium of human retinal pigment epithelial cells

**Figure 1:**
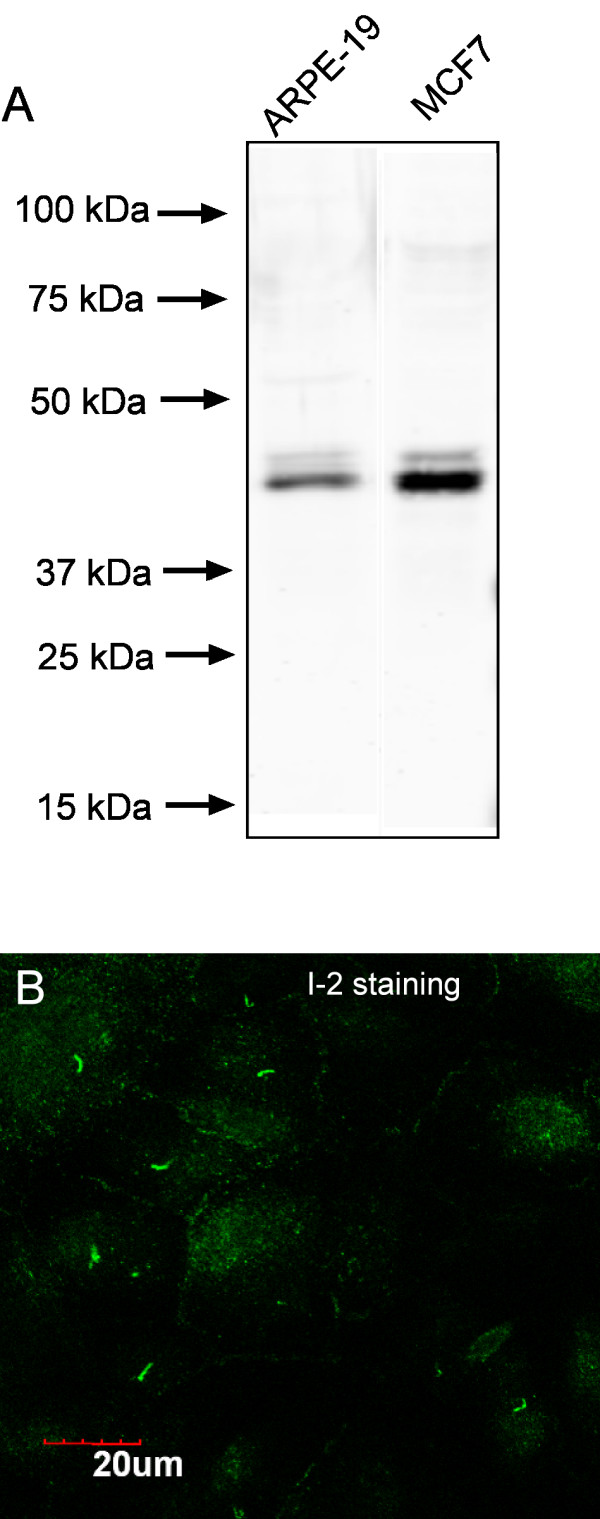
**I-2 localization by specific antibody staining in human ARPE-19 cells**. (A) Extracts from confluent ARPE-19 cells and MCF-7 cells were subjected to Western blotting using affinity-purified sheep antibody specific for human I-2. This antibody only recognized I-2, and its phosphorylated form (minor upper band) in both cell lines. (B) Confluent ARPE-19 cells were fixed, and stained with anti-I-2 antibody as described under Methods. Compressed Z-stack of optical sections from laser confocal microscopy is shown. Scale bar = 20 micron.

**Figure 2:**
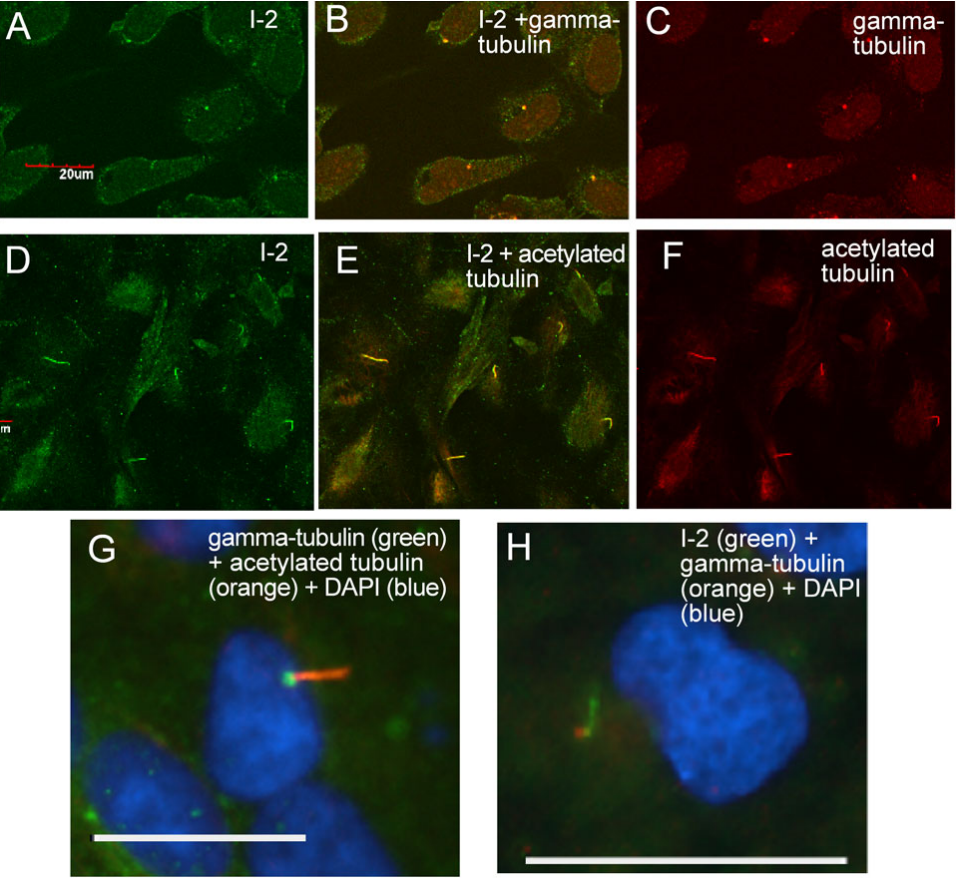
**Co-localization of I-2 and tubulin in ARPE-19 cells**. ARPE-19 cells at sub confluent density were fixed and stained for I-2 (A, green) and gamma-tubulin (C, red), and visualized by laser scanning confocal microscopy (merged image in B). Scale bar = 20 micron. ARPE-19 cells 48 hrs after confluence were fixed and stained for I-2 (D, green) and acetylated tubulin (F, red) with merged image in E. Compressed Z-stack of confocal optical sections is displayed to show the primary cilia. Scale bar = 20 micron. (G) Wide-field microscopic image to show localization of basal body (green, gamma-tubulin staining) and cilium (orange, acetylated-tubulin staining) in a confluent ARPE-19 cell. Scale bar = 25 micron. (H) Wide-field image to show the localization of endogenous I-2 (green), gamma-tubulin (orange) and nucleus (blue) in confluent ARPE-19 cells. Scale bar = 25 micron.

We found using laser confocal fluorescent microscopy that endogenous phosphatase inhibitor 2 (I-2) concentrated in the primary cilium of confluent human diploid retinal epithelial ARPE-19 cells (Fig. 1). I-2 was concentrated in one short segment that stained more brightly than the I-2 throughout the cytoplasm. Immunostaining of the cells used an affinity-purified antibody that detected a single protein corresponding to I-2 in whole cell extracts of either ARPE-19 or human breast cancer MCF-7 cells (Fig. 1A). A fraction of the I-2 in both cell lines was phosphorylated, as seen by a minor band of reduced mobility. In sub-confluent ARPE-19 cells endogenous I-2 co-localized with gamma-tubulin at the centrosome (Fig. 2A–C), as we previously reported [15]. The localization of I-2 was noticeably different after the cells became confluent (Fig. 2D–F). From the size, shape, and location, we suspected the brightly stained structure in each cell was the primary cilium, and confirmed this by co-staining for acetylated tubulin (Fig. 2D–F). In hundreds of cells, the cilia identified by anti-acetylated tubulin were all brightly co-stained by anti I-2. Further confirmation of these structures as primary cilia was based on co-staining for gamma-tubulin and for acetylated tubulin (Fig. 2G). The gamma-tubulin was localized at the basal body (green) that served as the base for the cilium, which contained acetylated tubulin (orange). Alternative double staining of gamma-tubulin and I-2 revealed the similar structure, with the gamma-tubulin (orange) appearing at the base of the cilium stained with anti-I-2 (green) (Fig. 2H). Our conclusion from these results was that phosphatase inhibitor 2 is localized at centrosomes in sub-confluent interphase ARPE-19 cells, and becomes concentrated in the primary cilium that extends from the centriole after cells reach confluence.

### Induction of primary cilium formation in ARPE-19 cells

**Figure 3:**
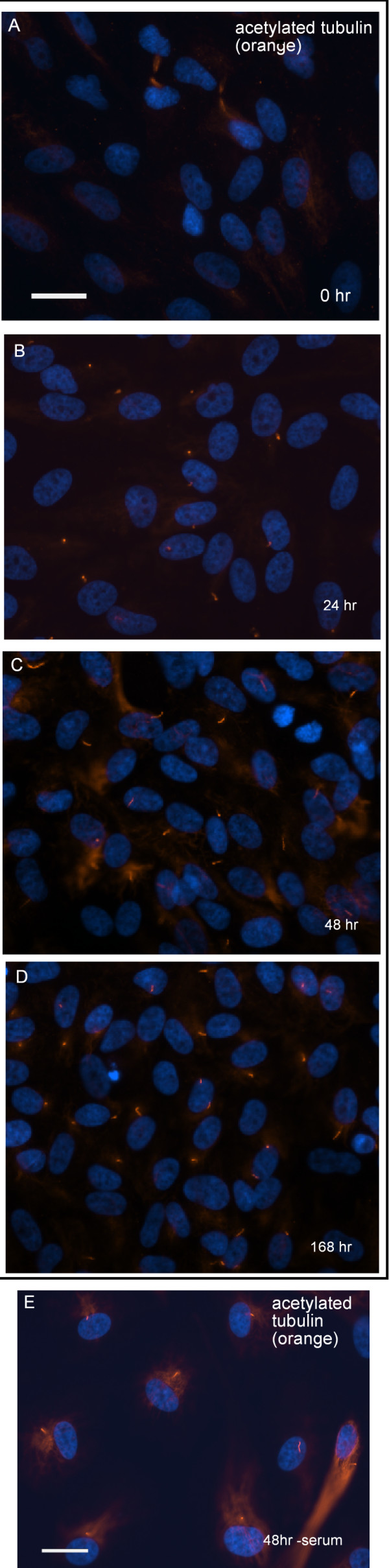
**Kinetics of primary cilium formation in ARPE-19 cells**. *(A – D)*. ARPE-19 cells at different time points (0 hr, 24 hrs, 48 hrs and 168 hrs) after confluence were fixed and stained for acetylated-tubulin (orange) and DNA (blue). Scale bar = 25 micron. (E) Sub-confluent ARPE-19 cells were incubated with serum free medium for 48 hrs, fixed and stained for acetylated-tubulin (orange) and DNA (blue). Scale bar = 25 micron.

To study how I-2 is involved in the primary cilium, we first examined the kinetics of cilium formation in ARPE-19 cells grown under different conditions (Figure 3). Confluent monolayers of cells were cultured in 10% FBS or serum-free medium for different periods of time, then fixed and stained for acetylated tubulin to identify the primary cilium. Acetylated tubulin staining was bright in the midbody of the few cells completing mitosis (Fig. 3A). At 24 hr post confluence, bright foci of acetylated tubulin that were not previously detected appeared in about 1/3 of the cells, indicating formation of a primary cilium (Fig. 3B). At 48 hrs post confluence (Fig. 3C), continuing up to 7 days thereafter (Fig. 3D), > 60% of cells formed a primary cilium that was brightly stained for acetylated tubulin. The kinetics of primary cilium formation in confluent ARPE-19 cells was not different whether the cells were incubated with or without 10% serum (data not shown). However, removal of serum from the medium induced formation of a primary cilium in sub-confluent cells (Fig. 3E).

### Detection of I-2 and PP1 in isolated primary cilia

**Figure 4:**
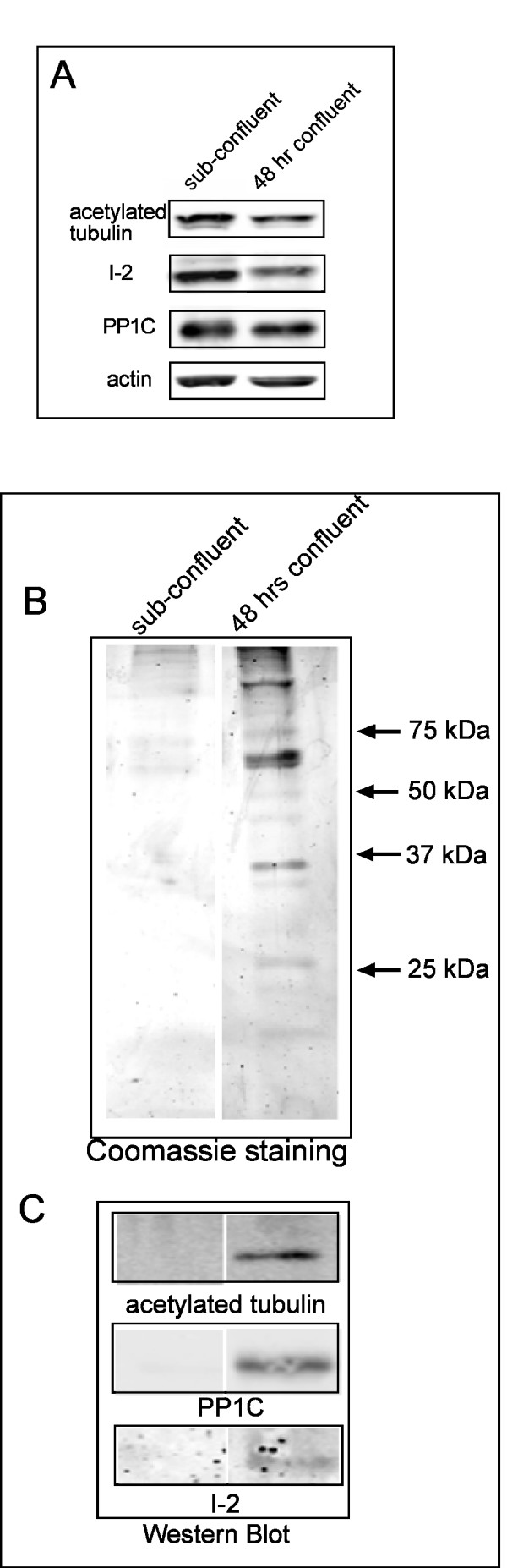
**Isolation and analysis of cilia fraction from ARPE-19 cells**. (A) Western blot to show levels of acetylated tubulin, I-2, PP1C and actin in equal numbers of sub-confluent and confluent cells used to isolate a cilia fraction. (B) Sub-confluent and 48 hr post confluent cells were used in parallel for cilia isolation by Calcium Shock and ultracentrifugation. Equal volumes of the cilia fraction were analyzed by SDS-PAGE in 12% gels stained with Commassie. (C) Western blotting to show the presence of I-2, PP1C and acetylated tubulin in the cilia fraction of sub-confluent cells and cells harvested 48 hrs after confluence.

We confirmed the localization of endogenous I-2 in the primary cilium by immunoblotting analysis of a cilia fraction dissociated from cells using the calcium shock method and recovered by differential ultracentrifugation. Equivalent numbers of sub-confluent and post-confluent cells were used as starting material in the cilia isolation procedure. Immunoblotting showed that there were identical amounts of actin and PP1 in these samples (Fig. 4A). Post-confluent cells had slightly lower levels of acetylated tubulin and I-2 compared to sub-confluent cells (Fig. 4A). The cilia fractions obtained from sub-confluent and post-confluent cells were resolved by SDS-PAGE, and proteins stained by Coomassie (Fig. 4B). Relatively few proteins were recovered in the cilium fraction from sub-confluent cells, whereas post-confluent cells yielded about a dozen of prominent proteins of different sizes. Proteins recovered in this cilia fraction represented selected enrichment of a subset of the proteins present in a whole extract of ARPE-19 cells. Immunoblotting identified acetylated tubulin, PP1C and I-2 in the cilia fraction from post-confluent cells that were not in the cilia fraction of sub-confluent cells (Fig. 4C). These results showed that acetylated tubulin, I-2 and PP1C were recovered in the primary cilium by biochemical fractionation, which reinforces the immunostaining of I-2 in the primary cilium of individual cells.

### I-2 concentrates in the primary cilium prior to tubulin acetylation

**Figure 5:**
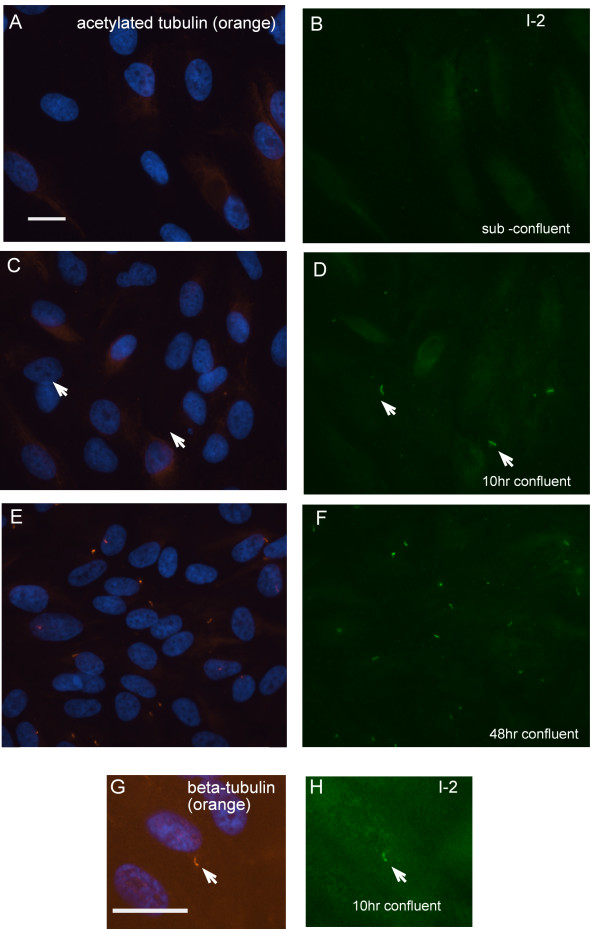
**Endogenous I-2 concentrates in the primary cilium before alpha-tubulin acetylation**. (A – F): ARPE-19 cells were fixed before confluence (A and B), 10 hrs after confluence (C and D), and 48 hrs after confluence (E and F), then stained for DNA (blue) + acetylated-tubulin (orange) (A, C and E) and co-stained for endogenous I-2 (green) (B, D and F). In C and D, white arrows point to the primary cilia where I-2 was present but acetylated tubulin was not detected. (G and H): ARPE-19 cells were fixed at 10 hrs after confluence, and triple stained for DNA (blue), beta-tubulin (orange) and endogenous I-2 (green). White arrow points to the primary cilia where both I-2 and beta-tubulin were present. Scale bars = 25 micron.

I-2 became concentrated in the primary cilium before the appearance of acetylated tubulin (Fig. 5). This was a curious observation, because tubulin acetylation is commonly used to identify the primary cilium. Sub-confluent cells showed diffuse cytoplamic distribution of acetylated tubulin and endogenous I-2 by double immunofluorescent staining (Fig. 5A and 5B). Hours after the cells became confluent, I-2 became concentrated into a structure that appeared to be the primary cilium, even though the structure was not co-stained for acetylated tubulin at this time (Fig. 5C and 5D). Later, after 48 hrs, confluent ARPE-19 cells displayed a primary cilium that co-stained for both I-2 and acetylated tubulin (Figure 5E and 5F). The structures that initially formed hours after confluence were co-immunostained for both I-2 and beta-tubulin (Fig. 5G, H) showing these indeed were primary cilia, even though they did not stain for acetylated tubulin. We concluded that I-2 was recruited into the primary cilium relatively early, preceding the accumulation of acetylated tubulin.

### I-2 is required for robust acetylation of tubulin in the primary cilium

**Figure 6:**
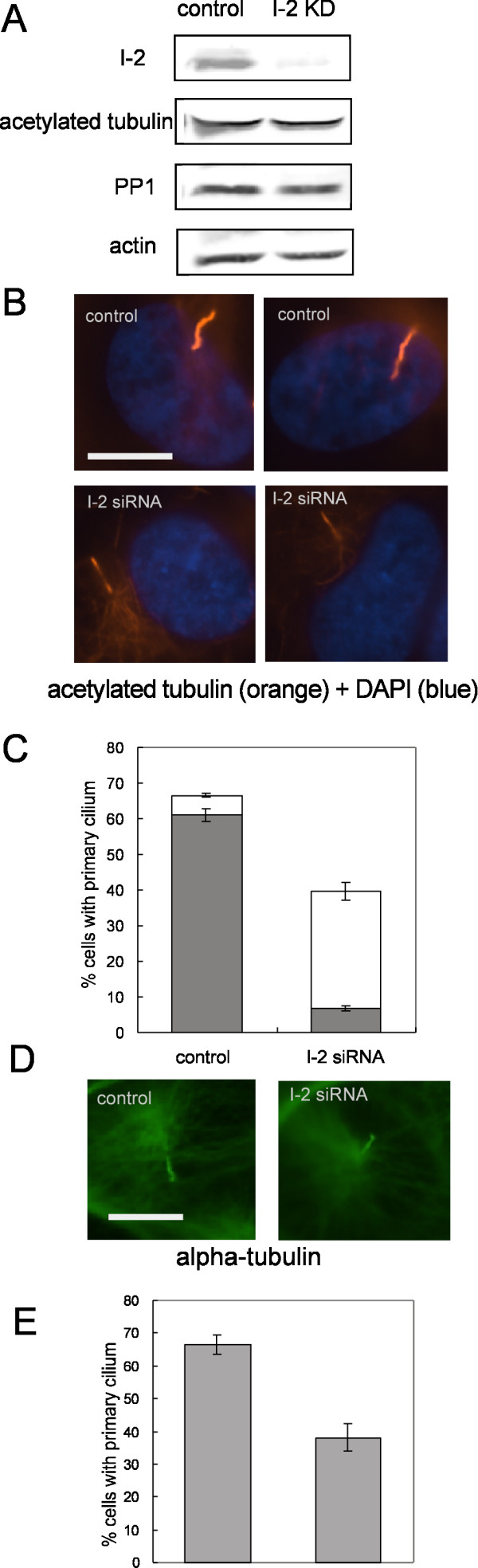
**Knockdown of I-2 reduces axonemal alpha-tubulin acetylation and primary cilium assembly**. (A) Cells were incubated with control siRNA or I-2 siRNA at 24 hrs before confluence, and collected 48 hrs after confluence. Extracts were subjected to immunoblotting as described in Methods. Actin was used as loading control. (B) ARPE-19 cells transfected with control siRNA or I-2 siRNA were fixed and stained for acetylated-tubulin (orange) and DNA (blue). Scale bar = 10 micron. (C) Quantitation of cells with a primary cilium. Cells with a primary cilium evidenced by acetylated-tubulin staining were scored in 200 cells per group. Intensity of tubulin acetylation in the primary cilium was measured as described in Methods. Solid bars represent percentage of cells possessing a primary cilium with full level of alpha-tubulin acetylation, whereas open bars represent percentage of cells possessing a primary cilium with reduced levels of tubulin acetylation. Data were plotted as mean ± SD of values from three independent experiments. (D) Cells transfected with control siRNA or I-2 siRNA were fixed and stained for alpha-tubulin (green). Scale bar = 10 micron. (E) Percentage of cells with a primary cilium based on alpha-tubulin staining was scored in 200 cells per group. Results were plotted as mean ± SD of values from three independent experiments.

We used siRNA transfection to knockdown the levels of endogenous I-2 to study effects on the primary cilium. Effective knockdown of I-2 required 24 hrs exposure to siRNA prior to confluence and I-2 protein levels were reduced by >85% compared to control siRNA transfected cells (Fig. 6A). I-2 knockdown did not change the levels of acetylated tubulin or PP1 in the cells, as determined by immunoblotting (Fig. 6A). We discovered knockdown of I-2 significantly reduced the fraction of cells with a primary cilium stained for acetylated tubulin, from 65% to 40% (Fig. 6C). Alternatively, staining for alpha-tubulin gave the same reduction in the fraction cells with a primary cilium due to knockdown of I-2 (Fig. 6D and 6E).

**Figure 7:**
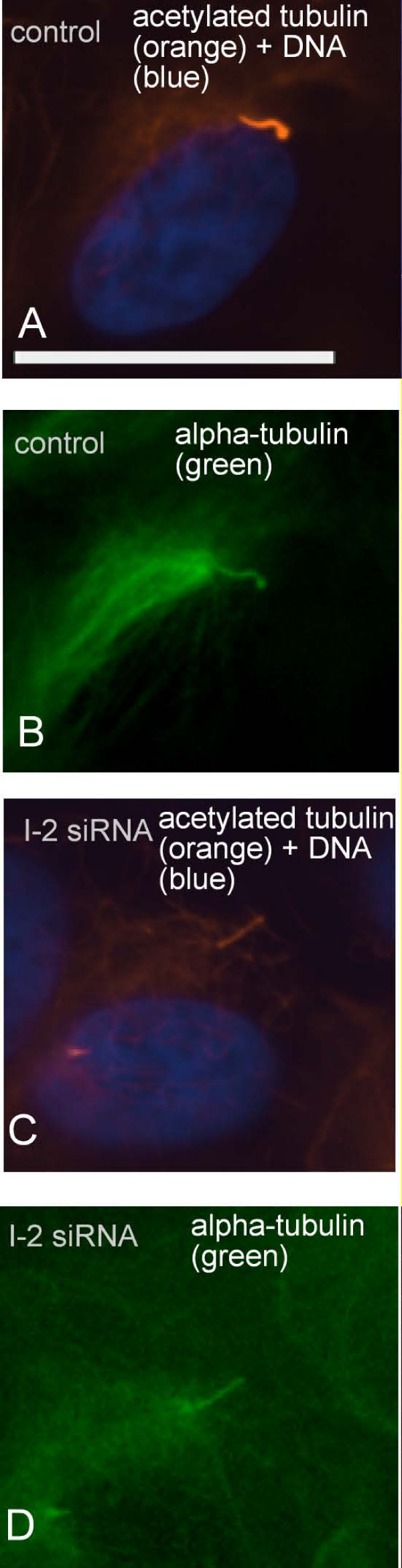
**Immunofluorescent staining of alpha-tubulin and acetylated tubulin in ARPE-19 cells**. ARPE-19 cells transfected with control siRNA or I-2 siRNA were fixed and triple stained for acetylated-tubulin (orange), DNA (blue) and alpha-tubulin (green). I-2 knockdown cells (C and D) showed reduction in acetylation of axonemal tubulin compared with control cells (A and B), without change in the staining of alpha-tubulin. Scale bar = 25 micron.

More striking than a reduction in the fraction of cells with a primary cilium was the reduction in the intensity of tubulin acetylation due to knockdown of I-2. When the axonemal tubulin was scored as either fully or partially acetylated there was nearly 90% reduction in cells with full tubulin acetylation in the primary cilium (Fig. 6B and 6C, solid bars). Thus, a major effect of siRNA knockdown of I-2 was a reduction in the level of tubulin acetylation in the primary cilium. This conclusion was verified by double immunofluorescent staining of control siRNA and I-2 knockdown cells for acetylated tubulin and alpha-tubulin (Fig. 7). Staining for alpha-tubulin was the same in control and I-2 knockdown cells (Fig. 7B, D), whereas the staining for acetylated tubulin was markedly reduced due to I-2 knockdown (Fig. 7A, C). I-2 knockdown did not decrease tubulin acetylation in either the cytosol of interphase cells or midbody of late mitotic cells (not shown). This argues that the effects of I-2 knockdown on tubulin acetylation were localized in the primary cilium, and not broadcast throughout the cell. We noted that the primary cilium in I-2 knockdown cells appeared to be approximately the same length as primary cilium in control cells (see both Fig. 6 and 7). The length of the primary cilium depends on the process of dynamic intraflagellar transport along the axonemal microtubules. We concluded that neither I-2 levels nor full tubulin acetylation were critical for these transport processes.

To demonstrate that the phenotype of reduced tubulin acetylation in the primary cilium was not due to the off-target effects of RNAi, we used another pair of siRNA targeting a different coding region of I-2. The knockdown efficiency and the phenotype were the same (data not shown). Alternatively, we combined siRNA transfection in sub-confluent ARPE-19 cells with induction of the primary cilium by serum starvation. In this protocol the primary cilium is formed without signals from cell-cell contacts that are formed during confluence. Knockdown of I-2 in sub-confluent cells significantly decreased the percentage of cells with a primary cilium and reduced tubulin acetylation in the primary cilium (not shown). Reduced tubulin acetylation by I-2 knockdown would be expected to reduce axonemal microtubule stability, and probably thereby result in the overall lower fraction of cells with a primary cilium.

### Reversal of I-2 knockdown phenotypes by inhibition of HDAC or PP1

We sought to obtain stringent proof of the specificity of RNAi by rescue of the knockdown phenotypes by over-expression of I-2, using an expression vector mutated to avoid RNAi. We over-expressed silently mutated I-2 in ARPE-19 cells, but found this severely reduced the percentage of confluent cells that formed a primary cilium (not shown). Therefore, we had to adopt alternative approaches to rescue the reduced acetylation of tubulin phenotype in I-2 knockdown cells.

**Figure 8:**
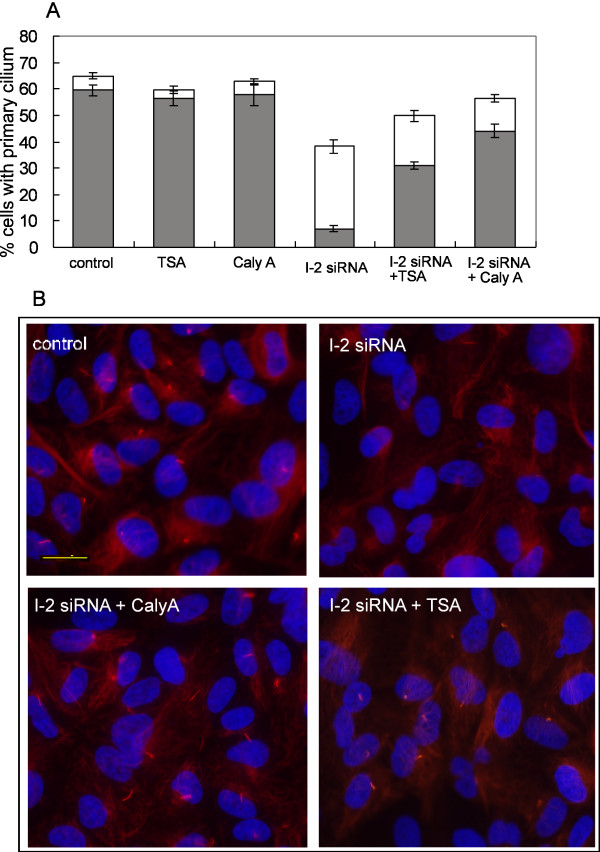
**Rescue of I-2 knockdown phenotype by chemical inhibition of PP1 or HDAC**. (A) ARPE-19 cells were transfected with siRNA as described in Figure 6. Cells were treated with 0.5 μM TSA or 0.5 nM calyculin A at 24 hr after siRNA transfection and 48 hrs later were fixed and immunostained for acetylated tubulin. Percentage of cells possessing a primary cilium with full or reduced levels of tubulin acetylation was counted as described in Figure 6. (B) Wide field images to show the acetylated tubulin staining (red) and DNA staining (blue) in cells treated with control siRNA, I-2 siRNA, I-2 siRNA + 0.5 nM calyculin A, or I-2 siRNA + 0.5 μM TSA. Scale bar represents 20 micron.

One approach was to inhibit multiple HDACs using the relatively non-specific inhibitor trichostatin A (TSA). Treatment with TSA partially restored to 50% the fraction of I-2 knockdown cells that formed a primary cilium, and had more dramatic and significant effect in increasing to > 30% the fraction of cells with full tubulin acetylation in the primary cilium (Fig. 8). Thus, inhibition of HDACs partially compensated for knockdown of I-2.

The other approach was to inhibit protein phosphatase type-1 (PP1) and type 2A (PP2A) using the cell-permeable compound calyculin A. Calyculin A is effective at nanomolar doses and shows some preference for PP2A over PP1 [21,22]. We tested a range of concentrations from 0.1 to 2 nM, and found that at 0.5 nM calyculin A there was partial rescue of I-2 knockdown cells in the fraction of cells with a primary cilium and of tubulin acetylation in the primary cilium (Fig. 8). Treatment of cells with the same doses of TSA or calyculin A without knockdown of I-2 produced no significant change in the fraction of cells with a primary cilium, and no change in tubulin acetylation in the primary cilium (Fig. 8). These results suggest that partial inhibition of PP1 was able to reverse the effect of I-2 knockdown.

## Discussion

This study shows that phosphatase inhibitor 2 (I-2) is concentrated in the primary cilium of human epithelial cells. We found endogenous I-2 localizes to the primary cilium prior to acetylation of the axonemal tubulin that serves as a marker for the primary cilium. Knockdown of I-2 by siRNA significantly suppressed the full acetylation of tubulin in the axoneme of the primary cilium, and reduced the percentage of cells with a primary cilium. These effects were rescued by pharmacological inhibition of PP1 or HDACs. Our results link I-2 and PP1, proteins involved in control of protein Ser/Thr phosphorylation, to the acetylation of tubulin in the primary cilium. This links two different systems of protein post-translational modification in the primary cilium.

Tubulin acetylation and deacetylation are catalyzed by a tubulin acetyltransferase and a specific histone/tubulin deacetylase (HDAC). Acetylation stabilizes microtubules [23], and the modification has been mapped to a single residue K40 of alpha-tubulin [24,25]. However, the identity of the acetyltransferase reactive with this site is still unknown. Among the multiple histone deacetylases, HDAC-6 has been shown to interact with and catalyze alpha tubulin K40 deacetylation [26,27]. HDAC-6 has been proposed to destabilize microtubules of the axoneme in the primary cilium and be regulated through phosphorylation involving Aurora A [9]. Previous results have shown that histone deacetylases, such as HDAC-1, HDAC-6, and HDAC-10 bind directly to the PP1 catalytic subunit [28,29]. Inhibition of HDACs by TSA causes dissociation of these HDAC-PP1 complexes, presumably through a conformational change. [28]. On the other hand, ATM dependent activation of PP1 by ionizing radiation led to dissociation of HDAC-PP1 complexes and dephosphorylation of HDAC-1, with an increase of HDAC activity [29]. Phosphorylation of I-2 directly by ATM is proposed to cause dissociation from PP1, accounting for PP1 activation by ionizing radiation [30]. Previous results therefore established functional links between PP1, HDAC-6 and I-2 and we suggest these are related to acetylation of tubulin in the primary cilium of epithelial cells.

PP1 was found in proteomic analysis of purified flagella from Chlamydomonas [31] and isolated human ciliary axonemes [32]. This supports the idea that PP1 regulates Ser/Thr phosphorylation of proteins in flagella and cilia. In addition, RT-PCR analysis showed that the levels of PP1 mRNA increased by 1.9-fold upon deflagellation, considered as evidence that PP1 is involved in flagellum function in Chlamydomonas. PP1 co-purifies with microtubules (MT) and binding to microtubules is mediated by the MT-associated protein Tau [33]. MT-associated phosphoproteins that might be PP1 substrates include kinesin and dynein motor complexes that are responsible for intraflagellar transport (IFT). However, the lack of change in size of the primary cilium in I-2 knockdown cells argues that I-2 does not regulate PP1 holoenzymes that control IFT. Altogether, various results point to some regulation of microtubule function and axoneme organization by PP1. In our experiments, inhibition of PP1 by 0.5 nM calyculin A rescued the reduction of alpha-tubulin acetylation in response to I-2 knockdown. Low doses of calyculin A are known to selectively inhibit PP2A [22,34,35], and we observed that they did not affect tubulin acetylation in the primary cilium in ARPE-19 cells (not shown). High doses of calyculin A were lethal to ARPE-19 cells. We surmised that the intermediate dose of calyculin A we used was producing selective but probably incomplete inhibition of PP1. We propose that I-2 affects tubulin acetylation and stabilization of the axoneme in the primary cilium by inhibiting the activity of specific PP1 holoenzymes. I-2 knockdown did not affect overall acetylation of tubulin, as detected by Western blotting whole cell extracts, or tubulin acetylation in either cytoplasm or midbody, as detected by immunostaining. We suspect that I-2 sensitive PP1 holoenzymes are specifically concentrated in the axoneme of the primary cilium to regulate ciliary alpha-tubulin acetylation, possibly by control of HDAC-6 activity. Previous work indicated that PP1 is targeted to microtubules by the protein Tau, however, Tau has not been identified by proteomics in flagella from Chlamydomonas or in axonemal fraction from cilia of human cells. This suggests that PP1 and therefore I-2 might be targeted to the primary cilium by some other PP1 regulatory subunit yet to be identified.

**Figure 9:**
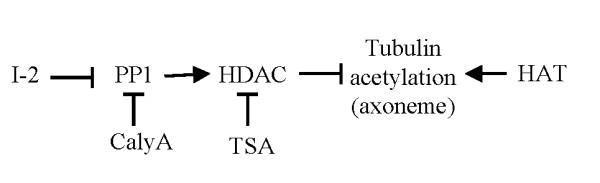
**Model for regulation of axonemal tubulin acetylation in the primary cilium by PP1 and I-2**. Axonemal tubulin acetylation is regulated by the balance of acetyltransferase (HAT) and deacetylases (HDACs). The tubulin-localized HDAC binds and interacts with MT-associated PP1. The activity of HDAC is negatively regulated by phosphorylation, so PP1 dephosphorylation produces activation. The presence of I-2 inhibits this PP1 holoenzyme and keeps the PP1-bound HDAC inactive. As a result the axonemal tubulin is highly acetylated and stabilized during formation of the primary cilium. When I-2 is depleted or dissociated from PP1-HDAC complex, PP1 dephosphorylates and activates HDAC, and results in ciliary tubulin deacetylation and destabilization of the axoneme of the primary cilium. Inhibition of PP1 by calyculin A or inhibition of HDAC by TSA can compensate for the function of I-2 in this pathway to increase tubulin acetylation.

Taken in sum, our data suggest a model (Fig. 9) for the regulation of tubulin acetylation by I-2 in the primary cilium of human epithelial cells. Axonemal tubulin acetylation is a balance between tubulin acetyltransferase and deacetylase. We propose that a HDAC forms a complex with a PP1 holoenzyme that binds I-2. I-2 inhibits the PP1 activity, keeping HDAC in an inactive, phosphorylated state. When I-2 is knocked down or dissociated from the PP1-HDAC complex, an increase of PP1 activity leads to dephosphorylation and activation of HDAC, favoring deacetylation of tubulin and destabilization of the primary cilium axoneme. This model invokes phosphorylation to negatively regulate HDAC-6, but the opposite has been suggested, i.e. HDAC-6 activation by phosphorylation. An alternative could be regulation of HDAC-6 by binding to PP1 with I-2 acting as an allosteric modifier of PP1 inhibition of HDAC-6. More detailed study of HDAC6-PP1 complexes should distinguish between these models.

Lastly, we have demonstrated unanticipated multifunctionality of I-2 that will need to be reconciled into more complex models. I-2 is a mitotic phosphoprotein substrate of CDK1-cyclin B1 [17,18]. In addition, I-2 binds and regulates the Pin1 prolyl isomerase [36]. Both Nek2A and Aurora A kinases are activated by I-2, the former indirectly by PP1 inhibition, the latter directly by protein-protein interaction [37,38]. In separate studies we have found that I-2 is required for proper chromosome segregation and cytokinesis, probably by indirect control of Aurora B [19]. Thus, I-2 has proved to be quite a versatile protein. The primary cilium may take advantage of I-2 to connect and coordinate different signaling pathways.

## Conclusion

Results of this study show that protein acetylation and phosphorylation are functionally interconnected to regulate the axonemal microtubules in the primary cilium. The regulatory protein I-2 localizes to the primary cilium prior to tubulin acetylation. Knockdown of I-2 by siRNA significantly reduced tubulin acetylation selectively in the primary cilium and chemical inhibitors of HDACs or PP1 partially reversed the effects of knockdown, showing that I-2 affects both phosphorylation and acetylation. As a mitotic phosphoprotein with multiple activities, I-2 may link cell cycle progression to the structure and function of the primary cilium.

## Methods

### Cell culture and transfection

Human adult retinal pigment epithelial cells (ARPE-19)(ATCC #CRL-2302) were grown according to ATCC recommendations. Cells were transfected with siRNA (80 nM) using Oligofectamine (Invitrogen) following the manufacturer' instructions. In the presence of serum, siRNA were incubated with cells 24 hrs prior to confluence to get efficient protein knockdown. Trichostatin A (TSA) was purchased from Sigma. Calyculin A was bought from Calbiochem. For the rescue assay, either 0.5 micromolar TSA or 0.5 nanomolar calyculin A was added into medium 24 hrs after siRNA transfection.

### siRNA preparation

Two pairs of siRNA targeting different coding regions of I-2 were designed using Dharmacon program , and ordered from Dharmacon (Lafayette, Colorado). The sequences of siRNA are available upon request.

### Immunoblotting

Western blotting as described [39] used the following primary antibodies: sheep polyclonal anti-I-2 (1:500); chicken anti-pan PP1 (1:20,000); mouse anti-actin (1:1000) (Sigma); mouse anti-acetylated tubulin (1:2000)(Sigma). Goat anti-rabbit Alexa Fluor 680, donkey anti-sheep Alexa Fluor 680 were purchased from Molecular Probes and Invitrogen and used at a 1:3000 dilution. Goat anti-mouse IRDye 800 and anti-chicken IRDye 800 antibodies were purchased from Rockland Immunochemicals and used at a 1:3000 dilution.

### Microscopy

Immunofluorescent microscopy was done as described [15] using the following primary antibodies: Sheep polyclonal anti-I-2 (1:100); Mouse anti acetylated-tubulin (1:500) (Sigma); FITC-conjugated anti-alpha-tubulin; mouse anti gamma-tubulin (1:1000) (Sigma); mouse anti beta-tubulin (1:50) (DSHB). Rhodamine Red-X-conjugated goat anti-mouse, Oregon Green-conjugated goat anti-rabbit or goat anti-sheep secondary antibodies were used at 1:1000 (Molecular Probes). DNA was stained with Hoechst 33342. Wide field images were obtained using Nikon Eclipse E800 microscope equipped with a Hamamatsu 3580 camera using OpenLab software 3.0. Confocal images were obtained using an Olympus FluoView™ FV 1000 system.

Acetylated tubulin staining in cilia was analyzed with the image-analysis software Openlab^® ^(Improvision, Coventry, UK). A mask (ROI) was created around acetylated tubulin staining in cilia. The mean pixel intensity of actylated tubulin staining in the masked region was corrected by subtracting background pixel intensity. A threshold of pixel intensity was set based on the comparison of staining intensity of control and I-2 knockdown cells. Axonemal tubulin in cilium whose acetylated tubulin staining intensity is above the threshold is counted as having normal tubulin acetylation, otherwise is counted as having inefficient tubulin acetylation.

### Isolation of primary cilia fraction from ARPE19 cells

Primary cilia were isolated by Calcium Shock technique as described [40].

## Abbreviations

(PP1): Protein phosphatase 1; (I-2): Phosphatase inhibitor-2; (TSA): Trichostatin A; (HDAC): Histone deacetylase

## Authors' contributions

WW cultured and stained cells, obtained microscopic images, did primary data analysis, designed experiments and drafted portions of the manuscript. DLB conceived of the project, participated in study design, analyzed data and wrote and edited the manuscript. Both authors have read and approved the final version of the manuscript.
